# Types of radical hysterectomies: From Thoma Ionescu and Wertheim to present day

**Published:** 2014-06-25

**Authors:** F Marin, M Plesca, CI Bordea, MA Moga, A Blidaru

**Affiliations:** * Department of Surgical Oncology II, "Prof. Dr. Al Trestioreanu" Institute of Oncology, Bucharest; ** "Dr. I.A. Sbarcea" Clinic Hospital of Obstetrics-Gynecology, Brasov

**Keywords:** radical hysterectomy, cervical cancer, surgical technique

## Abstract

Abstract

The treatment for cervical cancer is a complex, multidisciplinary issue, which applies according to the stage of the disease. The surgical elective treatment of cervical cancer is represented by the radical abdominal hysterectomy. In time, many surgeons perfected this surgical technique; the ones who stood up for this idea were Thoma Ionescu and Ernst Wertheim. There are many varieties of radical hysterectomies performed by using the abdominal method and some of them through vaginal and mixed way. Each method employed has advantages and disadvantages. At present, there are three classifications of radical hysterectomies which are used for the simplification of the surgical protocols: Piver-Rutledge-Smith classification which is the oldest, GCG-EORTC classification and Querlow and Morrow classification. The last is the most evolved and recent classification; its techniques can be adapted for conservative operations and for different types of surgical approaches: abdominal, vaginal, laparoscopic or robotic.

Abbreviations: GCG-EORTC = Gynecologic Cancer Group of the European Organization of Research and Treatment of Cancer; LEEP = loop electrosurgical excision procedure; I.O.B. = Institute of Oncology Bucharest; PRS = Piver-Rutledge-Smith

## Introduction

The treatment for cervical cancer is a complex, multidisciplinary one, which applies according to the stage of the disease. In the early stages, the treatment is represented by a simple intervention (LEEP or conization) having not only a diagnostic but also a therapeutic role, or by a simple total hysterectomy for women over 45, no longer willing to have children. In the advanced stages, the treatment is realized through neoadjuvant radiotherapy associated with the radical surgery [**4**]; as for the final stages, the treatment is no longer applied in a curative purpose, but a palliative one, the only therapeutic help being provided by an oncologist by radio- and chemotherapy.

 History

 The surgical elective treatment of cervical cancer is represented by the radical abdominal hysterectomy. This operation combines two conceptions: the conception of the organ extended surgery and the conception of the lymphatic territory surgery applied according to principle. The pioneers of the principles of radicality for the cervical cancer are the Viennese surgeon Ernst Wertheim and the Romanian surgeon Thoma Ionescu, who have sustained their view in 1902, at the International Congress of Surgery and Gynecology in Rome. By improving the technique proposed by Ries (1895) and Clark (1896), Wertheim proposes an organ surgery extended by the excision of the uterus, along with the surrounding conjunctive tissue, of the annexes and of the superior vagina; regarding the lymph node area, Wertheim suggested the only excision of the palpable lymph nodes. On the other hand, Thoma Ionescu was a strong supporter of the pelvic lymphadenectomy by principle, along with the excision of the uterus and its annexes; he established the delimitation between the operable cases and the inoperable cases, being a visionary of the stage classification of cervical cancer, which was set only after 35 years by Heyman. Because of the death rate and the great morbidness of the abdominal extended total hysterectomy, the extended surgery through the vagina developed more and more. This method was invented by Schauta (1901) and was later improved by Amreich. Moreover, the method does not correspond to the surgery of lymphatic territory and is criticized by many authors. In 1921, H. Okabayashi published his own technique, developed with his professor, S. Takayama, which had as particularity, the preservation of the nerve plexus [**5**]. In the mid 20th century, the curietherapy exceeded the radical surgery that had a significant death rate and morbidness. In 1944, Meigs improved Wertheim's technique and reported a survival rate of 75% for the patients diagnosed with first stage cervical cancer and an intraoperative death rate of less than 1%; thus, the radical surgery regained its popularity among the gynecological oncology. After a few years, Navratil (1950) and Subodh Mitra (1951) improved the vaginal hysterectomy method by adding the extraperitoneal ilio-obturator lymphadenectomy. As far as our country is concerned, in 1945, S. Vuia improved the technique of vaginal hysterectomies. With all these technique improvements the method was not imposed by principle, being practiced with exceptions. In 1956, the team made of I. Petroseanu, I. Chiricuta, Al. Trestioreanu and V. Mudric published among the pages of the Surgery magazine the extended lymphadeno-hysterocolpectomy, the so-called I.O.B. technique certified on thousands of cases, having exceptional results. Prof. Panait Sarbu, MD, and Prof. Dan Alessandrescu, MD, chiefs of clinic of two prestigious maternities in Bucharest, Giulesti and Polizu, have brought a significant contribution to the evolution of cervical cancer treatment.

 In 1974, Piver-Rutledge-Smith divided the radical hysterectomies into 5 classes, a classification respected by numerous surgeons and gynecologists. Nevertheless, over time, this classification became outdated and obsolete. In 2007, the Surgeons Committee of the Gynecologic Cancer Group, which was part of the European Organization of Research and Treatment of Cancer (GCG-EORTC) have proposed and adopted a new revised classification. Its purpose was to simplify and clarify some technique details, and in particular, to standardize the procedures in the oncology departments in Europe, that were taking part in the trials of this organization. In 2008, Querleu and Morrow published another classification of the radical hysterectomies in Lancet Oncology magazine, one that was different from the PRS or the GCG-EORTC classification and that was gaining more and more followers. It had as a particularity certain subtypes of the radical hysterectomy with the preservation of the autonomic nerves or with the paracervical lymphadenectomy.

**Table 1:**
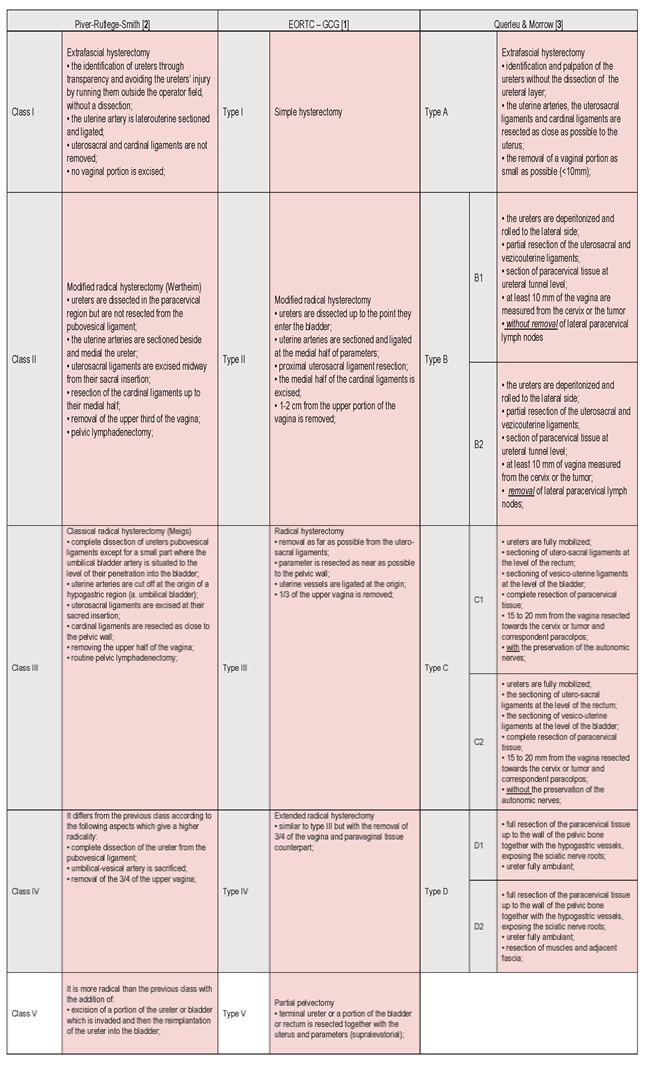
Classification of Radical Hysterectomies

**Fig. 1:**
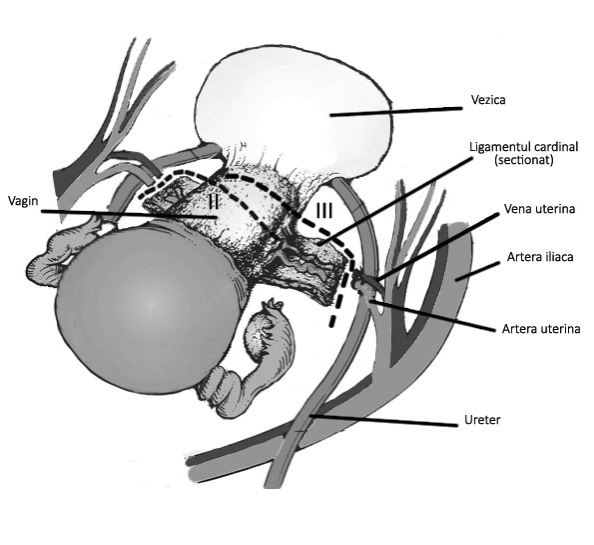
Difference between type II and type III radical hysterectomy (anterior view)

**Fig. 2:**
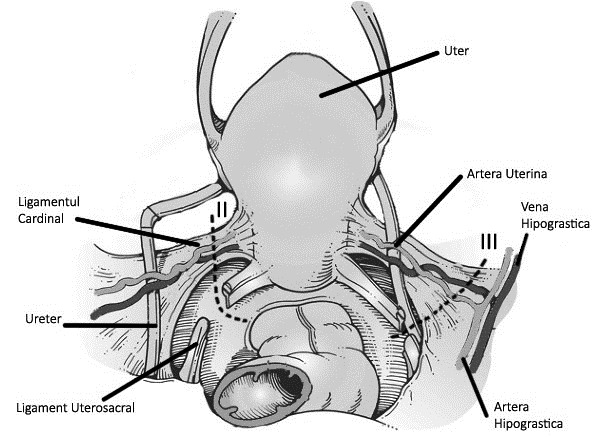
Difference between type II and type III radical hysterectomy (posterior view)

 Surgical abdominal techniques used in the cervical cancer

 Techniques that belong to the organ surgery: The simple total hysterectomy

 ∎ Disadvantages: misses possible cancerous areas (parameters, ganglion, superior vagina)

 Techniques which belong to the extended surgery: The extended Wertheim colpo-hysterectomy 

 Secondary techniques: 

 The extended total hysterectomy - J. L. Faure

 The extended total hysterectomy - Bonney

The extended total hysterectomy - Luros

 The extended total hysterectomy - Stanca

 ∎ Disadvantages: misses lymph nodes and the external part of the suburethral pedicles.

 Techniques that belong to the surgery of the system (organ extended surgery + lymph nodes tributary to the cervix surgery):

 ∘ Techniques that only associate the excision of the palpable lymph nodes with the extended hysterocolpectomy - Wertheim

 ∎ Disadvantages: misses ganglions that are not hypertrophied, but that can be invaded by cancer.

 ∘ Techniques that associate the excision of the "first" lymph node with the extended hysterocolpectomy - the Leveuf-Godard operation

 ∎ Disadvantages: misses the other lymph nodes, having as a starting point the fake premise that the first lymph node station is the obturatory node. 

 ∘ Techniques used to practice only the pelvic lymphadenectomy, the uterus and the parameters being missed, supposing that they were sterilized by the preoperative radiotherapy - the Taussig operation

 ∎ Disadvantages: the radiotherapy does not always sterilize the cervical lesion and the infiltration of the parameters.

 ∘ Techniques used to remove the entire lymph node tissue of the pelvis (with its 3 stations), along with the entire pelvic conjunctive tissue, in bulk with the uterus (annexes and vagina) - extended hysterocolpectomies 

 • The extended abdominal hysterocolpectomy with ilio-lombo-pelvic dissection (Thoma Ionescu)

 • The extended hysterocolpectomy - Meigs type

 • The extended hysterocolpectomies - Magendie and Brennier type

• The extended hysterocolpectomy - Brunschwig type

 • The extended hysterocolpectomies - Huguier and Magara type 

 ∎ Disadvantages: the total removal of the pelvic conjunctive tissue and of the aortico-cave lymph nodes (rarely invaded), with great postoperative sequelae and morbidness.

 ∘ Techniques used, along with the total colpo-hysterectomy to remove the lymph node tissue and the pelvic conjunctive tissue, due to the protection of the radiotherapy.

 • The extended lymphadeno-hystero-colpectomy technique - I.O.B. type

 • The M. Dargent technique

 ∎ Disadvantages: misses the parameters that are considered oncologically sterilized through radiotherapy.

 • The Wertheim technique associated with the systematic lymphadenectomy developed under the protection of the radiotherapy (Husslein)

 Surgical vaginal techniques in cervical cancer

∘ The Schauta-Stockel-Amreich technique, modified by S. Vuia

 ∎ Disadvantages: 

 - it does not provide the lymphadenectomy of the pelvis

 - it does not provide the evaluation of the abdominal organs

 - decreased operative field

 ∘ The Mitra technique associates a pelvic lymphadenectomy unique approach with the above mentioned technique, by approaching an abdominal extraperitoneal way (R. Michel Bechet, Ira Nathanson)

 ∎ Indications:

 - old women

 - cardiac

 - diabetic

 - obeseness

 - any situation that requires a spinal anesthesia

 Surgical mixed way (vaginal and abdominal) techniques 

 ∘ The Dan Alessandrescu technique

## Discussions

The surgical techniques in the treatment of cervical cancer have evolved according to the degree of radicalness, starting from simple operations which missed a lot of pelvic conjunctive tissue, possibly tumoral invaded, up to the supra-radical operations which were excising large portions of pelvic tissue and lymph nodes and had excessive death and morbidness rates [**7**]. This evolution developed along with the accumulation of experience on large groups of patients, who have been followed in time, with the purpose of discovering the tumoral recurrences. At the moment of the radiotherapy the radical surgery was introduced, interventions mediated the two tendencies; the postoperative complications became less significant, with a more acceptable death rate, with a long-term survival and a disease-free interval, all being more extended [**8**].

The Piver-Rutledge-Smith classification was conceived from the desire of elaborating protocols in the treatment of cervical cancer. It was noted that along with the evolution of the operative techniques, this type of classification had several deficiencies, becoming more and more ambiguous and obsolete. 33 years later, the Surgeons Committee of Gynaecologycal Cancer Group conceived a revised edition of the old classification, which was more practical and clinically significant due to the wish to standardize the clinical trials made within the European Organization of Research and Treatment in Cancer. Both classifications include type I, which is not a radical hysterectomy, but it is included due to its curative features in the treatment of initiative stages (0 and IA1-with any lympho-vascular invasion), IA2 and IB1 (the tumor having almost 1 cm). For these cancers, which was not very advanced, there was no need of the resection of the superior vaginal third like the PRS suggested, but only for a 1-2 cm resection. Type III of radical hysterectomy is indicated for the IB1 and IIA stages. Type IV differed significantly from class IV, PRS having the large cervical tumors IIA with borders of oncologic safety as an indicator. The partial exentaration (type V) is rarely used within the medical practice at present because it has a very high rate of morbidness and it was determined that the radio-chemotherapy has much better results. In the GCG-EORTC classification, the lymphadenectomy for the main groups of lymph nodes is indicated and appears in the end in N.B.1 for types II-V. According to this classification, the removal of the fallopian tubes and the ovaries is not part of the radical hysterectomy, being optional.

 In the Querleu and Morrow classification, not only the therapeutic effect of the techniques, but also the postoperative complications is taken into account [**9**]. To simplify, there are 4 types of radical hysterectomies which are described (A-D), but, when necessary, subtypes are being added, which take into consideration the paracervical lymphadenectomy and the preservation of the nerves [**10**]. The lymph nodes’ dissection on four stations is separated. The radical hysterectomies in this classification can be adapted for conservative operations (aiming for the procedure of fertilization) or in case of vaginal or abdominal open surgery, laparoscopic or robotic surgery.
